# How we stepped up to support others

**DOI:** 10.7554/eLife.98891

**Published:** 2024-04-29

**Authors:** Christina Elliott, Patrick Brundell

**Affiliations:** 1 School of Biomedical, Nutritional and Sport Sciences, Newcastle University Newcastle United Kingdom; 2 Mixed Reality Laboratory, University of Nottingham Nottingham United Kingdom

**Keywords:** sparks of change, research culture, being neurodivergent in academia, peer support, neurodiversity, neuroinclusivity

## Abstract

From diagnosis and disclosure to leading change, two neurodivergent researchers recount their experiences setting up peer support networks at their universities.

The pressures to ‘fit in’ can exact a heavy toll on neurodivergent people. Finding a supportive community that understands you and where you can truly be yourself can thus make a world of difference. In Sparks of Change, two academics share how they spearheaded grassroots initiatives to connect and empower their neurodivergent students and colleagues.

## Paying it forward for neurodivergent students

*Christina Elliott is a Lecturer in Biochemistry/Pharmacology at the School of Biomedical, Nutritional and Sport Sciences, Faculty of Medical Sciences, Newcastle University, Newcastle, United Kingdom.*

As a neurodivergent academic, self-disclosure felt akin to retiring as a spy. Not because I was engaging in any sinister deception, but because I had lived with a constant fear of being detected and the pressure to mimic the social behaviours of others to blend in and be safe. When I finally overcame my trepidations and shared my neurodivergence at work just over a year ago, to my surprise I found a warm acceptance as an ‘academic who came in from the cold’. This experience ignited my desire to make it easier for other neurodivergent people in academia – and especially students – to disclose without fear and to gain access to the support they need to thrive.

My early career had mostly been in laboratory research, where many of my ADHD traits felt like an advantage provided I also masked some of my other, more problematic traits to remain ‘under the radar’. I thrived on the daily novelty and dynamics of concurrent projects, and I could hyperfocus on my work with persistence and creativity. Moving between laboratories, cities and even countries every few years as a postdoc provided other opportunities for new experiences as well as self-reinvention.

That all changed when I finally settled in my current position as a primarily teaching-focused academic at Newcastle University. My tried-and-trusted coping strategies proved less effective in the face of the increased administrative burden and repetitive tasks (e.g., assessment marking!). My mask was slipping and I was struggling to maintain ‘my cover’.

Fortunately, I had a colleague who showed me it was possible to thrive as your whole self and stop living incognito. Damian Parry was well-versed in equality, diversity and inclusion matters; more importantly for me, he was also open within the university about being dyslexic. His example helped quell my concerns about disclosure, and he was the first person I told at work. His encouragement then led me to share with more colleagues and join a faculty staff network, which proved invaluable.

Disclosing my neurodivergence also led to adjustments that have improved my working life: for example, several meetings that I had to attend regularly were made shorter, and I moved from a crowded open-plan office to a quieter working space, to help escape constant distractions.

These experiences made me want to ‘pay it forward’ and become a visible role model for my neurodivergent students. I also came to recognise the importance of lowering the barriers around disclosure for other neurodivergent faculty, so that they too could become visible role models; because as Billie Jean King said, “if you can see it, you can be it”.

Whenever I am teaching, I have started to disclose my neurodivergence in the course introductions. This way my students can know what to expect, including why they may sometimes need to resend an email that I have missed. I now draw from my personal experiences when talking with and teaching neurodivergent students almost every day. Yet, I also felt motivated to do more.

Inspired by our positive experiences with the staff network, Damian and I pitched establishing a support network for neurodivergent students within our school. Following approval from key figures at the university, I took the lead on canvassing interest for the project and was transparent about my own neurodivergence throughout to help build trust with students concerned about disclosure.

We ended up launching our Neurodiversity Support Network in October 2023. The initiative provides neurodivergent students with social and academic support, both online and on campus. Most importantly, it specifically gives students the opportunity to come together with neurodivergent staff to share experiences, strategies and mentorship. Our students have found having visible neurodivergent staff members embedded as potential role models and mentors to be “amazing”, for both “aspirational reasons” and “for giving advice on things [they] have experienced”.

Our informal grassroots endeavour has now created a community that provided critical mass for advocacy to raise awareness and reshape teaching practices within the school and beyond (Box 1). The reaction from my institution has been surprisingly positive, and this framework will be rolled out more widely across the faculty. I am eternally grateful to those who inspired me to be brave and take charge of my neurodiversity narrative, for the benefit of my students, but also myself.

Box 1. **Advice for supporting neurodivergent students.**

1. Do not gatekeep by formal diagnosis. There are significant barriers to diagnosis, with some students waiting years even to be assessed. Allow students to join groups and networks based on self-identification.
2. Balance celebration with support. Students are often concerned with being tokenised and efforts therefore need to be made to provide meaningful support. Students commonly report being apprehensive about disclosure if they cannot see an immediate and tangible benefit.
3. Acknowledge the limits of your knowledge. Neurodiversity is a broad umbrella term, and neurodivergent people are not a monolith. Lived experiences even among neurodivergent people are variable, which can sometime lead to misunderstandings. Don’t expect yourself to know everything; always be willing to listen.
4. Review your approaches to inclusion. Neurodivergent students are often early indicators for non-inclusive practices. Designing your curriculum and tailoring your teaching approaches to meet the needs of neurodivergent students will likely confer wider benefits for the entire class.

## Making a space to be ourselves

*Patrick Brundell is a Research Fellow in the Mixed Reality Laboratory, University of Nottingham, Nottingham, United Kingdom.*

As I read the email, I sighed to myself. My excitement at a new member possibly joining our peer support network faded when I realised it was instead an academic leader with an ‘interest in neurodiversity’ asking to join despite not being neurodivergent themselves. While I could appreciate why they had likely got in touch, unfortunately (for them) this was not what the network was for.

When I had initiated the Neurodiversity Staff Network at the University of Nottingham in March 2021, it was during the splendid rush of research and self-empowerment following my own ADHD diagnosis about a year earlier. Like many, mine was a late diagnosis, and it had offered me an explanation for several of my past experiences; for example, why I frequently had bursts of intense creative energy at the beginning of new projects that were impossible to sustain, and why I had changed disciplines often, always seeking a new challenge.

Being diagnosed also made me eager to connect with people who might share similar experiences. Back then I didn’t know any other openly neurodivergent academics, but my partner was in the university’s Black, Asian and Minority Ethnic Staff Network, so I understood the difference a supportive community of minoritised people could make. I wanted to create a space where individuals like me could share their struggles and perhaps some keys to their successes, somewhere we could all feel seen and heard, away from the ‘neurotypical gaze’.

I remember growing increasingly impatient before the launch and somewhat frustrated with the many administrative tasks I had to do. Yet the university’s pro-vice-chancellor for equity, diversity and inclusion was supportive and had connected me with human resources, who took on some of the formalities of establishing the network. When we finally did launch and people began requesting to join, I was incredibly relieved.

The network was initially a virtual community over Microsoft Teams that we advertised as a peer support group for university staff at Nottingham who self-identified as neurodivergent. Immediately, however, we also became involved with a range of awareness-raising events across the university. Within months, for example, I’d begun teaching students about neurodiversity, openly discussing my experiences, and sitting on more committees than I could remember the name of. While not exactly why I had started the network, it all felt important work and was being encouraged by the university as part of a growing commitment to accommodating hidden disabilities.

Later, I managed to secure some internal funding to run workshops exploring the lived experience of neurodivergent staff and students. The aim was to identify how to support each other and where the university might improve its practices and structures. Creating a safe space seemed essential in fostering honest and open discussion, so only neurodivergent people were invited to take part.

People at the workshops repeatedly mentioned the value they got from this non-judgemental discussion of our joys and struggles. More than one person said regardless of any other outcome, the workshops had been like therapy. Someone cried as they recounted moving jobs every 18 months throughout their 30-year career as a familiar pattern of social rejection, confusion and ultimately withdrawal had played out over and over. Another explained how they had found their PhD to be the first challenging and exciting academic work they had ever done, discovering a passion for original research.

It also became apparent, however, that some of the network’s members didn’t take part in these events and had rarely attended or shared in our other meetings either. While accepting people could’ve joined for different reasons, there were concerns that – like those looking to join with just an ‘interest in neurodiversity’ – some existing members might be motivated by professional curiosity rather than personal experiences.

Those concerns compromised the perceived safety of the space we had set out to create. The network’s committee therefore implemented some changes to allay those fears. We formally asked all members to agree that the network was solely for people who self-identify as neurodivergent. We also clarified that we existed to provide a safe space for discussion and support rather than satisfy curiosity about neurodiversity, and we renamed ourselves accordingly.

These decisions were not made to downplay the important role of neurotypical colleagues in fostering a neuroinclusive working environment. Rather, they felt like the right way to ensure we could create a place where individuals could express their true self freely, and which best served the people the network had been set up to support. Importantly, while these changes did lead a few members to step away, there was already a separate staff group called Neurodiversity at Nottingham that welcomed anyone interested in networking and sharing information, neurodivergent and neurotypical alike.

Today, our renamed Neurodivergent Staff Network boasts a membership of 150, with meetings regularly drawing over 40 members. Looking back, I’m proud of what we have achieved together. While I believe collaborating with university structures does hold promise, for me the network’s true value lies in us supporting each other and sharing our experiences; I hope this will continue.

Finally, to any neurodivergent researcher embarking on establishing a peer support network within their institution: remember that the systemic shifts needed for large organisations to reduce inequity will take longer than the neurodivergent desire for ‘everything now’ can easily cope with. Prioritise looking after yourself and your members in the short term; your organisation can take care of itself.

## About this article

This Sparks of Change article is part of a series on being neurodivergent in academia, which includes a list of tips, resources and tools collated by neurodivergent scientists.

